# 25 Jahre „International Classification of Functioning, Disability and Health“: Was Forschung und Praxis voneinander lernen können

**DOI:** 10.1007/s00103-026-04274-y

**Published:** 2026-07-09

**Authors:** Anke Scheel-Sailer

**Affiliations:** Zentrum für Rehabilitation und Sportmedizin, Inselgruppe, Berner Reha Zentrum, Universität Bern, Schwendi 299, 3625 Heiligenschwendi, Schweiz

**Keywords:** Funktionsfähigkeit, Rehabilitation, Implementierung, Partizipation, ICF, Functioning, Rehabilitation, Implementation, Participation, ICF

## Abstract

Dieser Diskussionsbeitrag gibt einen Überblick über die Nutzung der Internationalen Klassifikation der Funktionsfähigkeit, Behinderung und Gesundheit (ICF) in Rehabilitation, Forschung, Bildung, Politik und Teilhabeplanung.

Die ICF wurde 2001 publiziert, um eine Grundlage für die Beschreibung von Funktionsfähigkeit und Einschränkungen der Funktionsfähigkeit von Menschen mit Behinderungen oder chronischen Erkrankungen im biopsychosozialen Modell zu schaffen. In den vergangenen 10 Jahren wurde die Nutzung insbesondere aus der Perspektive der Forschung für die Praxis diskutiert.

Die ICF findet Anwendung in der medizinischen Versorgung, Forschung, Bildung und Politik. Eng verbunden mit der Gesundheitsstrategie Rehabilitation trägt sie zu einem besseren Verständnis von Rehabilitation bei. Beispielhaft zu nennen sind hier die Aktivitäten der Weltgesundheitsorganisation, die Entwicklung von ICF Core Sets zur Beschreibung der Funktionsfähigkeit bei ausgewählten Krankheitsbildern, die Integration des ICF-Konzeptes in Leitlinien sowie nationale und internationale Implementierungsprojekte. Auch in zahlreiche Ausbildungen ist das Konzept der ICF integriert; ergänzend werden Weiterbildungsmodule angeboten. In der sozialen Eingliederung und beruflichen Reintegration hat die Nutzung der ICF zu einer leistungsrechtlichen Teilhabeplanung und einer ICF-orientierten Bedarfsermittlung beigetragen.

Auch wenn sich die ICF in allen geschilderten Bereichen als Konzept bewährt hat, besteht weiterhin eine Lücke zwischen in der Forschung entwickelten Demonstrationsprojekten und ihrer flächendeckenden Nutzung. Häufig zeigen diese Projekte eine Komplexität und einen Aufwand, die eine konsequente Anwendung im Alltag erschweren. Politische Unterstützung und Weiterentwicklungen in der Forschung und der Gesellschaft auf Grundlage der bisherigen Erfahrungen sind notwendig, um Funktionsfähigkeit als Gesundheitsindikator sowie als Grundlage für die soziale Integration und Teilhabe flächendeckend zu etablieren.

## Abstract (en)

The International Classification of Functioning, Disability and Health (ICF) was published in 2001 to establish a framework for describing the functioning and limitations in functioning of people with disabilities or chronic conditions within the biopsychosocial model. The use of the ICF has been discussed over the past decade from the perspective of research for practice.

The ICF has been used in the fields of medical care, research, education, and policy. Closely linked to the health strategy of rehabilitation, the ICF has contributed to a better understanding of rehabilitation. Examples include the activities of the World Health Organization, the ICF Core Sets as a basis for describing functioning of selected clinical health conditions, the integration of the ICF concept into guidelines, and national and international implementation projects. The ICF concept has been integrated into numerous training courses, and continuing education modules are offered. In the areas of social inclusion and vocational reintegration, the use of the ICF has led to participation planning based on entitlement to benefits and ICF-oriented needs assessment.

Although the ICF has proven itself as a concept in all the areas described, there remains a gap between demonstration projects developed in research and the widespread use of the ICF. Demonstration projects often reveal a level of complexity and effort that makes consistent use in everyday life difficult. Political support and further developments in research and society, based on the experiences gained, are necessary to establish the tool’s effectiveness as a health indicator and to promote social integration and participation on a nationwide scale.

## Einleitung

Mit der Veröffentlichung der Internationalen Klassifikation der Funktionsfähigkeit, Behinderung und Gesundheit vor 25 Jahren im Jahr 2001 (ICF; [1]) hat die Weltgesundheitsorganisation (WHO) ein Modell lanciert, welches in Ergänzung zur Internationalen Klassifikation der Diagnosen (ICD) Menschen nicht nur im Hinblick auf die Diagnosen, sondern auch in ihrer Funktionsfähigkeit beschreiben soll. Ziel war es, diese ICF in allen Ebenen des Gesundheitssystems und der WHO zu nutzen. Die ICF sollte den Menschen in seiner Funktionsfähigkeit, seiner Aktivität und Partizipation in der Interaktion mit der Umgebung in den Mittelpunkt stellen [2]. Seit 2005 steht die ICF in deutscher Sprache in gedruckter Form und seit 2020 auf den folgenden Internetseiten 1 zur Verfügung. In dem Praxisleitfaden ICF der Bundesarbeitsgemeinschaft für Rehabilitation (BAR) e. V^2^ in der 2. Auflage 2016 wurden Schwerpunkte wie die Entwicklungen im gesellschaftlichen/politischen Bereich (z. B. Inklusion, UN-Behindertenrechtskonvention (UN-BRK)), Ansätze von Fachgesellschaften zur fehlenden Ausgestaltung der Komponente der personbezogenen Faktoren und die vielfältigen Erfahrungen in der Nutzung der ICF in der Praxis der Rehabilitation dargestellt.

Das Ziel dieses Diskussionsbeitrages ist es, einen Überblick über internationale und nationale Aktivitäten im Hinblick auf die Nutzung der ICF aus der Perspektive der Forschung und über den Transfer in die Praxis und die Gesellschaft zu geben, um zukünftige Strategien für eine nachhaltige Nutzung der ICF zu diskutieren.

## WHO und Rehabilitation

Die ICF wurde in den vergangenen Jahren von der WHO einerseits für die Weiterentwicklung der Rehabilitation als Gesundheitsstrategie und andererseits für die soziale Integration genutzt. Erwähnt sei hier der „Global Disability Action Plan 2014–2021 and Rehabilitation 2030: A Call to Action“ durch die WHO [3]. Mit der Verabschiedung der Resolution zur Stärkung der Rehabilitation im Gesundheitswesen auf der 76. Weltgesundheitsversammlung am 27.05.2023 hat die WHO der Rehabilitation als Gesundheitsstrategie eine Schlüsselrolle gegeben und sie auf die Ebene eines Menschenrechts gestellt [4].

Die WHO weist darauf hin, dass in der sich verändernden Gesellschaft und Demografie mit der älter werdenden Bevölkerung eine von 3 Personen mit Gesundheitseinschränkungen lebt, die von Rehabilitation profitieren würde. Die WHO beschreibt Rehabilitation umfangreich und inklusiv:

„Rehabilitation addresses the impact of a health condition on a person’s everyday life by optimizing their functioning and reducing their experience of disability. Rehabilitation expands the focus of health beyond preventative and curative care to ensure people with a health condition can remain as independent as possible and participate in education, work and meaningful life roles. Anyone may need rehabilitation at some point in their lives, whether they have experienced an injury, disease, illness, or because their functioning has declined with age“.^3^

Die WHO hat mehrere Arbeitsgruppen zur Förderung der Rehabilitation als Gesundheitsstrategie initiiert und interprofessionell und interdisziplinär „Packages of interventions for rehabilitation“ mit verschiedenen Schwerpunkten erstellt.^4^ Aus dieser gemeinsamen Arbeit hat sich in der Folge die „World Rehabilitation Alliance“ interprofessionell und interdisziplinär gebildet, welche sich auf Rehabilitation als eine wesentliche Intervention im „Universal Health Coverage“ fokussiert.^5^ Zusammenfassend ist Rehabilitation und damit auch die ICF in der WHO konzeptionell und exemplarisch sichtbar verankert.

## Initiativen medizinischer Fachgesellschaften zur Nutzung der ICF

Die Internationale Gesellschaft für Physikalische Medizin und Rehabilitation (ISPRM) hat sich nach ihrer Gründung 1999 inzwischen zu einer weltweit vernetzten Fachgesellschaft entwickelt, mit mehreren Tausend Mitgliedern und mehr als 30 nationalen Fachgesellschaften als aktive Mitglieder in der ISPRM 2026.^6^ Die ISPRM arbeitet in der WHO-Liaison eng mit der WHO zusammen.

Ergänzend arbeiten nationale oder internationale Fachgesellschaften oder Spezialisten an der konzeptionellen Umsetzung und Vorbereitung der Integration der Rehabilitation und zur Nutzung der ICF. So hat zum Beispiel die Sektion der Europäischen Union der Spezialisten für Physikalische Medizin und Rehabilitation (UEMS-PRM) in den letzten Jahren Konzepte zur Beschreibung der Rehabilitation [5] und einen Plan zur Stärkung der Rehabilitation erarbeitet [6]. Es wurden die Klassifikation von Rehabilitationsangeboten (International Classification of Services in Rehabilitation: ICSO-R 2.0; [7]), der klinische „Assessment Schedule“ (CLAS; [8]) und das individuelle Rehabilitationsprojekt (IRP; [9]) beschrieben. Neben der strukturierten Beschreibung der Funktionsfähigkeit wurde der Zielsetzungsprozess mit kurz-, mittel- und langfristigen Zielen im Hinblick auf eine gelingende Partizipation als ein Kernelement in der Rehabilitation definiert [10].

Basierend auf diesen konzeptionellen Entwicklungen in den letzten 10 Jahren wird in der Folge ein Beispiel eines Implementierungsprojektes vorgestellt. In diesem wird deutlich, wie die ICF als Grundverständnis in der Rehabilitation genutzt werden kann, Leitlinien auf dieser Basis entwickelt und Rehabilitationsangebote biopsychosozial ausgerichtet werden können, um eine größtmögliche Partizipation in der Gesellschaft vorzubereiten.

## Praxisbeispiel: Implementierung der ICF

Als ein Beispiel werden die wissenschaftlich begleitete Implementierung einer ICF-basierten Rehabilitation bei Menschen mit einer Querschnittlähmung auf der individuellen, der institutionellen Gesundheitsversorgung und die Rahmenbedingungen für die Umsetzung der ICF-basierten Rehabilitation auf nationaler Ebene beschrieben. Als Grundlage werden die oben beschriebenen Konzepte genutzt.

### Implementierung einer ICF-basierten Rehabilitation für Menschen mit Querschnittlähmung

In einer vorbereitenden Situationsanalyse zeigte sich, dass die ICF-basierte Rehabilitation grundsätzlich in das interdisziplinäre und interprofessionelle Management integriert wurde, aber eine ICF-basierte Datenerhebung der Funktionsfähigkeit noch nicht möglich war [11]. Obwohl standardisierte Gesundheitsinformationen aus narrativen Berichten extrahiert werden konnten, erforderte dieser Prozess Zeit und Fachwissen und war für den Alltag nicht realistisch [11]. Anstatt ICF-Codes direkt aus der klinischen Routine zu erstellen, dienten daher Assessments und Untersuchungen während der Erstrehabilitation nach Eintritt einer Querschnittlähmung als Ausgangspunkt für die ICF-basierte Beschreibung der bereits routinemäßigen und standardisiert erfassten Funktionsfähigkeit [12, 13]. In der klinischen Praxis wurden während der Erstrehabilitation von Patienten und Patientinnen mit Querschnittlähmung Assessments der sensomotorischen Funktionen, der Mobilität und der Selbstversorgung eingesetzt, während andere Assessments des autonomen Nervensystems, der Schmerzen, der Teilhabe oder der Lebensqualität fehlten [13].

Auf der Grundlage dieser Beobachtungen und der nationalen und internationalen Anforderungen sollte ein Standard für ein Assessment-Toolkit allgemein als auch für bestimmte Untergruppen definiert und umgesetzt werden [13]. Im Rahmen eines Leitlinienprojekts der Deutschsprachigen Gesellschaft für Paraplegiologie (DMGP) für „Ergebniserhebung in der Erstbehandlung nach neu erworbener Querschnittlähmung“ und auf der Grundlage einer systematischen Übersichtsarbeit [14] empfahl die Leitliniengruppe Assessments, die die für die Querschnittlähmung relevanten Strukturen und Funktionen sowie Aktivität, Teilhabe und Lebensqualität beschreiben. Die empfohlenen Assessments waren wissenschaftlich fundiert und ICF-basiert zur Definition eines Standards für Ergebniserhebungen nach Querschnittlähmung [15].

Der Nottwil-Standard wurde in einem Querschnittzentrum als Standard entwickelt, um diese ICF-basierte Leitlinie unter Einbezug von Kohortenstudien und nationalen Qualitätsanforderungen in den klinischen Alltag zu implementieren [16]. Er umfasst 10 Untersuchungen, 23 Assessments und 2 Fragebögen. Insgesamt werden 55 ICF-Kategorien beschrieben, darunter die meisten Kategorien des ICF Generic-30 Set [16]. Die erfolgreiche Implementierung war möglich, weil sich die Geschäftsleitung engagierte, ein Multiprojektmanagement den Umsetzungsprozess begleitete, Richtlinien für die Dokumentation erarbeitet, ein manuelles Kontrollsystem etabliert und die Schulung der Mitarbeiter zum Nottwil-Standard geplant wurden [16]. Der gesamte Entwicklungs- und Implementierungsprozess dauerte knapp 10 Jahre und kann als Hinweis auf die Komplexität gewertet werden.

Um die ICF-basierte Rehabilitation auf nationaler Ebene zu konzeptualisieren, diente das europäische Framework für Rehabilitationsangebote als Ausgangspunkt [17]. Vertreter von 12 nationalen medizinischen Fachgesellschaften, einer nationalen interprofessionellen Rehabilitationsgesellschaft, die Schweizer Vertreter von 2 internationalen Rehabilitationsgesellschaften und die Leiter der 4 Querschnittzentren nahmen an einem mehrstufigen Konsensprozess teil, um ein Framework für Rehabilitationsangebote für Querschnittlähmung in der Schweiz zu definieren („SCI/D Framework“; [18]). Die daraus resultierende Version des „SCI/D Framework“ umfasst 19 Rehabilitationsangebote, die in 9 Cluster gegliedert sind, von denen 6 in allgemeine, andere spezifische oder spezifische Rehabilitationsangebote für Querschnittlähmung unterteilt sind. Für die Klassifikation von Rehabilitationsangeboten konnte ICSO‑R 2.0 verwendet werden, um ein Querschnittzentrum in der Schweiz für 2 Rehabilitationsangebote (postakut und ambulant) zu beschreiben [19]. Trotz einiger Herausforderungen könnte die ICSO‑R 2.0 die nationale Berichterstattung und Zertifizierung ermöglichen, die den internationalen Anforderungen entspricht [19].

### „Lessons learned“

Die Implementierung einer ICF-basierten Rehabilitation bei Querschnittlähmung ist möglich, wenn die Mikro‑, Meso- und Makroebene berücksichtigt werden. Wissenschaftlich durchgeführte Situationsanalysen, die Methoden wie Interviews, Fokusgruppen und Kohortenstudien anwenden, waren bei der Entwicklung eines maßgeschneiderten Qualitätsverbesserungsprozesses zur Implementierung einer ICF-basierten Rehabilitation hilfreich. Nationale und internationale Anforderungen sowie der konzeptionelle Rahmen dienten auf der einen Seite als Grundlage für das Projekt und förderten auf der anderen Seite das Engagement der am Projekt beteiligten Akteure. Der nutzerzentrierte Ansatz im Konsensprozess erhöhte die Motivation und verringerte den Widerstand gegen den Veränderungsprozess. Diese Einstellung förderte den kontinuierlichen Verbesserungsprozess. Durch eine angemessene Ressourcen- und Zeitplanung wurden Frustrationen und ein frühzeitiger Ausstieg aus dem Projekt vermieden.

## Nutzung der ICF in der Forschung

Nachdem die Nutzung der ICF 2012 kritisch diskutiert und praxisorientierte Implementierung und Forschung empfohlen wurde [20], konnten in einem Review 2025 das kontinuierliche Interesse und die Fokussierung auf einen biopsychosozialen und personenzentrierten Ansatz gezeigt werden [21]. Es zeigte sich eine Zunahme der Publikationen um ca. 13 % jährlich seit 2011 vor allem aus den Ländern USA, Kanada, Deutschland, den Niederlanden und der Schweiz, häufig aus den Forschungsgruppen der Maximilians-Universität München, der Schweizer Paraplegiker-Forschung Nottwil und der McMaster-Universität. Neben dem Fokus Rehabilitation kristallisierte sich in dem Review heraus, dass in den letzten Jahren Themen wie Epidemiologie, Altern und gesundheitsbezogene Lebensqualität im Kontext der ICF im Vordergrund standen.

Die Unterstützung zur Weiterentwicklung der Forschung zur ICF zeigte sich auch in Organisationen wie der ICF Research Branch^7^ und den „World Health Organization Collaborating Centers of the Family of International Classifications (WHO-FIC CCs)“. In einer kürzlich durchgeführten Umfrage über die Nutzung der ICF im nationalen Kontext zeigte sich, dass die ICF hauptsächlich in der klinischen Praxis, in der Politikentwicklung und Sozialpolitik, weniger häufig im Bildungsbereich und in der Forschung eingesetzt werde. Trotz ihrer Anwendungen in verschiedenen Sektoren sei die Verwendung der ICF in den meisten Ländern nicht verpflichtend. Wo sie eingesetzt werde, biete sie einen biopsychosozialen Rahmen für die Politikentwicklung in den Bereichen Gesundheit, Funktionsfähigkeit und Behinderung [22]. Das Spannungsfeld zwischen Implementierung eines konzeptionellen Verständnisses und der vergleichbaren und messbaren Implementierung der Funktionsfähigkeit stellt wahrscheinlich auch heute noch eine Herausforderung dar.

Mit der Gründung von Cochrane Rehabilitation am 22.10.2016 wurde ein weiterer Meilenstein für die wissenschaftlich fundierte Beschreibung der Rehabilitation erreicht. Rehabilitation wird als „multimodal, person-centered, collaborative process“ (Intervention-general), including interventions targeting a person’s „capacity (by addressing body structures, functions, and activities/participation) and/or contextual factors related to performance“ (Intervention-specific) with the goal of „optimizing“ the „functioning“ (Outcome) of „persons with health conditions currently experiencing disability or likely to experience disability, or persons with disability“ (Population) beschrieben [23]. Ziel war es, eine Brücke zwischen Cochrane und Stakeholdern aus dem Bereich Rehabilitation zu schaffen. Es wurde definiert, wie die ICF als Referenzsystem für eine vergleichende Evaluation und eine standardisierte Beschreibung von Rehabilitationsinterventionen genutzt werden soll. Dies würde beinhalten, ICF-Kategorien für Interventionsstudien im Bereich Rehabilitation zu definieren, spezifische Kategorien und die dazugehörigen Assessments zu definieren und Funktionsfähigkeit standardisiert zu berichten. Hunderte von systematischen Reviews konnten inzwischen nach den Cochrane-Regeln publiziert werden [23].

### Linking ICF

Ein wesentlicher Meilenstein in der Nutzung der ICF wurde durch die erstmalig 2005 [24] veröffentlichten und 2019 überarbeiteten „Linking Rules“ gelegt [25]. In mehreren Hundert Publikationen wurde diese Methode angewendet, um einerseits das Konzept der ICF als bereits im Alltag genutzt aufzuzeigen und andererseits die Grundlage für Vergleichbarkeit von Rehabilitationsinterventionen durch eine strukturierte Beschreibung der Funktionsfähigkeit zu schaffen.

Als Beispiel sei hier auf 2 systematische Reviews verwiesen [7, 26], in denen das ICF-Linking genutzt wurde, um die Ergebnisse von Rehabilitationsinterventionen nach einem zerebrovaskulären Ereignis in der akuten und der chronischen Phase in ICF-Kategorien zu beschreiben. Die Outcome-Parameter umfassten in den Studien Körperfunktionen (*n* = 19), gefolgt von Aktivitäten und Teilhabe (*n* = 14), Umgebungsfaktoren (*n* = 4) und personbezogenen Faktoren (*n* = 2). Auch wenn die Outcome-Parameter gut zur ICF gelinkt werden konnten und damit die Verbesserung der Funktionsfähigkeit in allen Studien gezeigt wurde, war die ICF nicht immer explizit erwähnt worden. Eine kombinierte Nutzung von diagnosebezogenen Informationen und Informationen zur Funktionsfähigkeit könnte ein nächster Entwicklungsschritt sein [26].

### ICF-basierte Core Sets

Ein wichtiger Meilenstein nach Publikation der ICF im Hinblick auf die Nutzung der ICF war die Entwicklung der ICF-basierten Core Sets. In den letzten 10 Jahren wurden weltweit ICF-basierte Core Sets zur Beschreibung wichtiger Krankheitsbilder entwickelt [28] und reevaluiert.^8^ Ein generisches Core Set als minimal oder für die Rehabilitation für die Beschreibung der Funktionsfähigkeit wurde entwickelt, um die Beschreibung der Funktionsfähigkeit zu vereinheitlichen und in den Alltag zu implementieren [29]. Um die Nutzung zu vereinfachen, wurden einfache und intuitive Beschreibungen entwickelt, die in Pilotprojekten getestet wurden [30, 31]. Obwohl sich in diesen Pilotprojekten vielversprechende Ansätze zeigten, konnte sich eine flächendeckende Nutzung im medizinischen Spannungsfeld noch nicht durchsetzen.

### Perspektive klinischer Nutzen

Im Bereich der Rehabilitation hat sich das Konzept für die Begleitung von Menschen mit chronischen Erkrankungen und Einschränkungen der Funktionsfähigkeit konzeptionell weltweit durchgesetzt. Die Erfassung der ICF-basierten Funktionsfähigkeit zu Beginn und zum Abschluss einer Rehabilitationsmaßnahme hat sich in nationalen Qualitätsinitiativen etabliert. Häufig werden aber nur einzelne Bereiche der Funktionsfähigkeit insbesondere im Bereich der Aktivitäten abgebildet. Fehlende validierte Messinstrumente insbesondere in den Schwerpunkten Umgebungsfaktoren und personbezogene Faktoren schränken eine konsequente umfangreiche Nutzung ein.

In einer Beobachtungsstudie wurde die ICF als integraler Bestandteil eines umfassenden und multimodalen Ansatzes in der Beschreibung der Funktionsfähigkeit vor und nach einer kardialen Rehabilitation genutzt [32]. Mit dem Konstrukt der ICF-Codes und der ICF-Qualifier konnten Unterschiede in der Funktionsfähigkeit der beiden Krankheitsbilder aufgezeigt werden. Aufgrund des retrospektiven Studiendesigns und der fehlenden Dokumentation konnten allerdings einige Kategorien des „ICF Maugeri Cardiological Set“ nicht eingeschlossen werden. Ebenso konnten die personbezogenen Faktoren und die Umgebungsfaktoren noch nicht erfasst und müssten erst in zukünftigen prospektiven Studien integriert werden [32]. Auch im Bereich der Neuroonkologie konnten die Assessments aus klinischen Studien zu den ICF-Kategorien gelinkt werden, auch wenn die Erfassung der Interventionen noch nicht durch die internationale Klassifikation der Gesundheitsinterventionen abbildbar war [33].

### Integration von ICF in Kohortenstudien

Ein Beispiel einer ICF-basierten prospektiven Kohortenstudie ist die „Swiss Spinal Cord Injury Cohort Study“ (SwiSCI; [34]), in der in einem wissenschaftlich fundierten und interdisziplinär und interprofessionell geführten Prozess die relevanten Themen für Menschen mit einer Querschnittlähmung definiert wurden und die Funktionsfähigkeit abgebildet und gemessen werden sollte [35]. Im Kontext dieser nationalen Kohortenstudie entstanden zahlreiche Publikationen, die auf dem Boden eines biopsychosozialen Modells in der Klassifikation der ICF die gelebte Erfahrung von Menschen mit einer Querschnittlähmung untersuchten und Zusammenhänge aufzeigen konnten [36]. Neben vielen anderen Aspekten konnte damit die soziale Teilhabe [37], die berufliche Eingliederung [38] als auch der Einfluss von Umgebungsfaktoren [39] bei Menschen mit einer Querschnittlähmung untersucht werden. Die Erfahrungen dieser Kohortenstudie wurden in einem nächsten Schritt genutzt und führten zu einer groß angelegten internationalen Kohortenstudie für Menschen mit einer Querschnittlähmung, in der das Konzept der ICF als Grundlage für die Beschreibung der Funktionsfähigkeit genutzt wurde [40]. Besonders sei hier auch auf die Möglichkeiten der nationalen Perspektive im internationalen Austausch hingewiesen und die Möglichkeit, Empfehlungen für die Weiterentwicklung von Versorgungsstrukturen geben und auf die Gesundheitspolitik wirken zu können [41]. Diese ICF-basierte Beschreibung der Funktionsfähigkeit und damit Teilhabe von Menschen könnte als nationales ICF-orientiertes Datensystem genutzt und zur Entwicklung von Disability-Indikatoren im Sinne der Eingliederungshilfesteuerung verknüpft werden.

### Implementierung der ICF in Leitlinien

Mit den zunehmenden wissenschaftlichen Publikationen auf der Basis der ICF und dem Wissen um eine Nutzung der ICF im klinischen Alltag tauchte das Konzept der ICF auch in Handlungsempfehlungen verschiedener Leitlinien auf. Dort wird es als Grundkonzept genutzt oder zu ICF-Codes gelinkt [42–44]. Auch wenn die ICF in einigen Leitlinien genutzt wird, taucht das Konzept in anderen Leitlinien noch nicht auf, sodass sich diesbezüglich eine flächendeckende Implementierung erst im Verlauf zeigen wird.

## ICF in Aus- und Weiterbildung

Während das Konzept der ICF in zahlreichen Ausbildungen, wie zum Beispiel dem Bachelor im Bereich der Therapien, integriert wurde und damit auch eine Grundlage für ein interprofessionelles Management darstellt [45], wird das Konzept der ICF nur sporadisch im Medizinstudium angeboten. Studierende schildern zunehmend, dass sie von der Behandlungsstrategie Rehabilitation eher gehört haben, ICF aber noch kein fester Bestandteil war.

In Rehabilitationskliniken und assoziiert an Forschungsgruppen etablierten sich interprofessionell ausgerichtete Weiterbildungsangebote in Kleingruppen oder als Onlinekurse und wurden von den Teilnehmenden sehr wertgeschätzt [46]. Beispielhaft sei hier auf die ICF-Schulungen an der LMU München^9^ und den Online-Kurs „ICF-StARS“ der Schweizer Paraplegiker-Forschung (SPF) und der Universität Luzern verwiesen.^10^

## Erfassung der Funktionsfähigkeit auf nationaler Ebene

Für die Implementierung einer ICF-basierten Beschreibung der Funktionsfähigkeit wäre die Nutzung bereits vorhandener Daten ein erster Schritt. In einer Studie wurde das Vorhandensein regelmäßig gesammelter Daten über die Funktionsfähigkeit in der Schweiz untersucht [47]. Dabei wurden 4 Datenquellen identifiziert: Schweizerische Umfrage zu Gesundheit, Alterung und Ruhestand in Europa (SHARE), die Schweizerische Gesundheitsbefragung (SHS), die Lausanne-Kohorte 65+ (Lc65+) und das Schweizer Haushalt-Panel (SHP). Alle Instrumente befassten sich mit Schlaffunktionen, Energieniveau, emotionalen Funktionen, Schmerzempfinden und 9 zusätzlichen Kategorien der Funktionsfähigkeit. In einem Scoping-Review wurden nationale Erfassungssysteme zur Beschreibung der Funktionsfähigkeit als „Disability Indicator“ gesucht. Aufgrund der fehlenden Daten zur Beschreibung der Funktionsfähigkeit auf nationaler Ebene war eine Vergleichbarkeit noch nicht möglich [48].

## Sozialrechtliche Aspekte

### ICF in der Eingliederungshilfe Bedarfsermittlungsverfahren

Mit dem Bundesteilhabegesetz (BTHG) wurde in § 13 SGB IX^11^ festgelegt, dass der Rehabilitationsbedarf der leistungsberechtigten Person individuell sowie ganzheitlich zu ermitteln ist (Leistungen aus einer Hand).^12^ § 118 Abs. 2 SGB IX ermöglicht es den Ländern, mittels einer Rechtsverordnung Näheres zum Bedarfsermittlungsinstrument zu regeln. Mit der ICF-Orientierung bei der Bedarfsermittlung wurde die Abkehr vom Fürsorgesystem hin zu einer leistungsrechtlich kodifizierten Teilhabeplanung möglich.

Im Spannungsfeld zwischen normierter Indikatorenbildung und individueller Lebenswelt muss die Bedarfsermittlung im kommunalen/landesrechtlichen Bedarfsermittlungsverfahren neu durch ein Instrument erfolgen, das sich an der ICF orientiert (§ 118 Abs. 1 SGB IX). Der Umsetzungsstand der Bedarfsermittlungsinstrumente § 118 SGB IX in den Bundesländern ist im Dokument Umsetzungsstand nachzulesen.^13^ In verschiedenen Bundesländern, z. B. Thüringen, Berlin, Niedersachsen, Westfalen-Lippe, wurden im Projekt Umsetzungsbegleitung BTHG Praxisbeispiele mit unterschiedlichem Fokus, wie z. B. der Integrierte Teilhabeplan, Einbezug des Sozialraumes, ein einheitliches Instrument zur Bedarfsermittlung oder Teilhabeplanung im Bereich Arbeit, entwickelt.

### ICF in der beruflichen Rehabilitation/Teilhabe am Arbeitsleben

Die Deutsche Vereinigung zur Rehabilitation (DVfR) hat konkrete Empfehlungen zur ICF-Nutzung im Sinne der ICF als biopsychosoziales Strukturmodell, für Leistungsbemessung, Teilhabeziele, Verlaufsdokumentation erarbeitet.^14^ Grundlage ist das Gesetz zur Stärkung der Teilhabe und Selbstbestimmung von Menschen mit Behinderungen (Bundesteilhabegesetz – BTHG). Im Praxisleitfaden der Bundesarbeitsgemeinschaft für Rehabilitation (BAR-Praxisleitfaden 4)^15^ wurde die ICF zur Bedarfserkennung, Bedarfsfeststellung, Teilhabeplanung und Dokumentation in beruflichen Reha-Einrichtungen genutzt. In der konkreten Ausarbeitung und Implementierung zeigten sich immer wieder Spannungen zwischen dem rehabilitationsmedizinischen Funktionsbegriff und den arbeitsmarktpolitischen Zumutbarkeits- und Verfügbarkeitserfordernissen (z. B. ICF-basierte Beschreibung von Leistungsfähigkeit vs. rentenversicherungsrechtliche Erwerbsminderungsprüfung), sodass eine weitere Nutzung forschend unterstützt und mit den beteiligten Stakeholdern diskutiert werden sollte.

## Diskussion und Ausblick

In diesem Update über die Nutzung der ICF in den letzten Jahren konnte gezeigt werden, dass das Konzept der ICF in der Begleitung oder Beschreibung von Menschen mit chronischen Erkrankungen und Einschränkungen der Funktionsfähigkeit weltweit genutzt wird. Die Wirksamkeit von Rehabilitationsmaßnahmen wird in vielen Ländern im Rahmen der ICF gemessen oder kann zu ICF-Kategorien gelinkt werden. Das Konzept der ICF ist in zahlreichen Leitlinien verankert und in zahlreiche Ausbildungen integriert worden. Interprofessionell ausgerichtete Weiterbildungsangebote in Kleingruppen oder als Onlinekurse können niederschwellig genutzt werden, um Wissenslücken zu schließen. Das Konzept der ICF wird auch in sozialrechtlichen Aspekten wie Eingliederungshilfe und der beruflichen Wiedereingliederung genutzt.

Trotzdem wird in zahlreichen Publikationen und Projekten geschildert, wie schwierig eine flächendeckende Nutzung der ICF ist, wie viele Menschen im Gesundheitssystem weder das Konzept der ICF noch der Rehabilitation als Gesundheitsstrategie kennen. Erst wenige medizinische Fakultäten in Deutschland und der Schweiz haben einen Lehrstuhl für Rehabilitation und bieten strukturierte Weiterbildungen zu diesem Thema im Studium an. Auch wenn das Konzept der ICF in zahlreichen Ländern in die Gesetzgebung integriert ist, werden Daten zur Funktionsfähigkeit noch nicht konsequent für die Beschreibung der Funktionsfähigkeit von Menschen mit chronischen Erkrankungen oder Behinderungen genutzt. Als Gründe für die zögerliche Implementierung werden die fehlende politische Entscheidungsbereitschaft, die fehlenden Konzepte zur flächendeckenden Erfassung der Funktionsfähigkeit und die noch nicht ausgereiften Modelle zur Integration der Komplexität im Einzelnen und der Vergleichbarkeit im Gesundheitssystem diskutiert.

Aus diesen Ausführungen wird deutlich, dass es sich bei der Implementierung um einen vielschichtigen kulturellen und konzeptionellen Prozess auf unterschiedlichsten Ebenen im Gesundheitssystem und in der Gesellschaft handelt, der den seit mehreren Jahrzehnten andauernden Wandel erklären kann. So wie die Nutzung der ICD nur einen Aspekt der Entwicklungen von Gesundheitssystemen darstellt, ist die ICF eine Klassifikation, die nicht einfach in das Gesundheitssystem integriert werden kann. Die oben aufgeführten Projekte und Entwicklungen bestätigen deutlich, dass das Konzept der Funktionsfähigkeit sinnvoll ist, auch wenn nicht einfach zu messen. Neben den etablierten Verfahren der Rasch-basierten Umrechnung werden moderne statistische Verfahren, wie z. B. Item-Response-Theorie, zu einer Verstetigung der ICF beitragen [49]. Forschungsschwerpunkte zum Thema Epidemiologie der Funktionsfähigkeit werden notwendig sein. Politische Unterstützung und ein Interesse an der Funktionsfähigkeit des Einzelnen und der Gesellschaft können ein Motor im Hinblick auf eine systematische Erfassung der Funktionsfähigkeit und eine valide Darstellung der Veränderung der Funktionsfähigkeit sein. Die Zufriedenheit auf der Seite der Gesundheitsfachpersonen und der Forschenden wird in diesem komplexen Veränderungsprozess eine Energiequelle sein. In zahlreichen Arbeiten wurde deutlich, dass die Zufriedenheit von Menschen mit Einschränkungen der Funktionsfähigkeit steigt, wenn sie in ihrer umfassenden biopsychosozialen Individualität wahrgenommen und begleitet werden [50]. Für dieses Ziel lohnt es sich, die immer wieder auftretenden Spannungen zwischen Klinikern, Politikern und Wissenschaftlern zu überwinden und aus den verschiedenen Perspektiven das Thema Nutzung der ICF weiterzuverfolgen.

## Data Availability

Alle dieser Arbeit zugrunde liegenden Daten sind in diesem Artikel enthalten.

## Footnotes

1 http://www.dimdi.de/static/de/klassi/icf/; https://apps.who.int/classifications/icfbrowser/ 14.04.2026

2 https://www.bar-frankfurt.de/fileadmin/dateiliste/_publikationen/reha_grundlagen/pdfs/BroschuereICF4_13.pdf 14.04.2026

3 https://www.who.int/health-topics/rehabilitation#tab=tab_1. Letzter Zugriff am 14.04.2026.

4 https://www.who.int/teams/noncommunicable-diseases/sensory-functions-disability-and-rehabilitation/rehabilitation/service-delivery/package-of-interventions-for-rehabilitation. Letzter Zugriff am 14.04.2026.

5 https://www.who.int/initiatives/world-rehabilitation-alliance. Letzter Zugriff am 14.04.2026.

6 https://isprm.org/natsoc/. Letzter Zugriff am 14.04.2026.

7 https://icf-research-branch.org/. Letzter Zugriff am 14.04.2026.

8 https://www.icf-core-sets.org/. Letzter Zugriff am 14.04.2026.

9 https://ihrs.ibe.med.uni-muenchen.de/veranstaltungen/icf_schulung1/index.html. Letzter Zugriff am 23.04.2026.

10 https://www.openlearnity.org/courses/StARS/. Letzter Zugriff am 23.04.2026.

11 https://www.sozialgesetzbuch-sgb.de/sgbix/118.html. Letzter Zugriff am 13.04.26.

12 https://umsetzungsbegleitung-bthg.de/beteiligen/fd-bedarfsermittlungsinstrumente-in-der-anwendung/. Letzter Zugriff am 13.04.26.

13 https://umsetzungsbegleitung-bthg.de/w/files/fachdiskussionen/umsetzungsstand-c-118-sgb-ix.pdf. Letzter Zugriff am 13.04.26.

14 Umsetzung des Gesetzes zur Stärkung der Teilhabe und Selbstbestimmung von Menschen mit Behinderungen (Bundesteilhabegesetz – BTHG) analysiert und unterbreitet konkrete Vorschläge zur ICF-Nutzung. Letzter Zugriff am 14.04.2026.

15 https://www.bar-frankfurt.de/fileadmin/dateiliste/_publikationen/reha_grundlagen/pdfs/BroschuereICF4_13.pdf. Letzter Zugriff am 14.04.2026.
